# Potential Protective Effect of Dietary Intake of Non-*α*-Tocopherols on Cellular Aging Markers Mediated by Tumor Necrosis Factor-*α* in Prediabetes: A Cross-Sectional Study of Chinese Adults

**DOI:** 10.1155/2020/7396801

**Published:** 2020-05-15

**Authors:** Yiwen Liu, Chifa Ma, Pingping Li, Chunxiao Ma, Shuli He, Fan Ping, Huabing Zhang, Wei Li, Lingling Xu, Yuxiu Li

**Affiliations:** ^1^Department of Endocrinology, Key Laboratory of Endocrinology, Ministry of Health, Peking Union Medical College Hospital, Peking Union Medical College, Chinese Academy of Medical Sciences, Beijing 100730, China; ^2^State Key Laboratory of Bioactive Substance and Function of Natural Medicines, Institute of Materia Medical Sciences and Peking Union Medical College, Beijing 100050, China; ^3^Diabetes Research Center of Chinese Academy of Medical Sciences, Beijing 100050, China; ^4^Department of Nutrition, Peking Union Medical College Hospital, Beijing 100730, China

## Abstract

It remains unknown how different glucose tolerance status affects the relationships between dietary intake of different tocopherol isoforms (*α*-, *β*-, *γ*-, and *δ*-tocopherol) and cellular aging, oxidative stress, and inflammatory markers. The authors conducted a cross-sectional study among 582 Chinese adults with different glucose tolerance status to explore the association between dietary intake of different tocopherol isoforms and cellular aging, oxidative stress, and inflammatory markers. The inverse correlations between non-*α*-tocopherols and tumor necrosis factor-alpha (TNF-*α*) varied substantially across different glucose tolerance status, with the strongest observed in prediabetes (*r* = −0.33 for *β*-/*γ*-tocopherol, *r* = −0.37 for *δ*-tocopherol, *p* < 0.01), followed by normal glucose tolerance (NGT). While such correlations were abolished in established diabetes. Furthermore, within prediabetes, the strongest inverse correlations between non-*α*-tocopherols and TNF-*α* were observed in impaired fasting glucose (IFG) (*r* = −0.42 for *β*-/*γ*-tocopherol, *r* = −0.55 for *δ*-tocopherol, *p* < 0.01), while such correlations were significantly attenuated in individuals with impaired glucose tolerance (IGT) and IFG+IGT. And mediation model analysis displayed that TNF-*α* mediated the protective effect of non-*α*-tocopherols on leukocyte telomere length and mitochondrial DNA copy number, which was uniquely observed in prediabetes, while such mediation effect was statistically nonsignificant in NGT and established diabetes. In conclusion, our findings indicate that dietary intake of non-*α*-tocopherols might protect against cellular aging markers mediated by TNF-*α* in prediabetes. Individuals with prediabetes, especially for IFG, might benefit from increasing dietary intake of non-*α*-tocopherol in alleviating inflammation and cellular aging, which might provide a new dietary avenue for delaying diabetes onset.

## 1. Introduction

Tocopherols (Toc), with four isoforms including *α*-, *β*-, *γ*-, and *δ*-Toc in diet, are potent peroxyl radical-scavenging antioxidants and anti-inflammatory agents [1]. *α*-Toc was the most studied because of its abundance in the diet and circulation, followed by *γ*-Toc, while *δ*-Toc was scarcely investigated. And increasing evidence has indicated that non-*α*-Toc isoforms appear to have superior antioxidant and anti-inflammatory properties compared to *α*-Toc [1–3], which may provide new important physiological and pharmacological discoveries that are useful for prevention and therapy against chronic diseases. Oxidative stress and inflammation participate in the cellular aging process [4, 5] as well as age-related diseases [6]. Given their antioxidant and anti-inflammatory effects, Toc potentially protect against the cellular aging process as well as age-related diseases [7]. Numerous studies have explored the effect of Toc on the product of DNA oxidation, i.e., 8-hydroxy-2deoxyguanosine (8-OHdG), and yielded mixed results [7–9]. However, few investigations explored the associations between Toc and cellular aging markers such as leukocyte telomere length (LTL) [10] and mitochondrial DNA copy number (mtDNAcn) [11], both of which were hypersensitive to oxidative stress and inflammation [12, 13]. As one of the age-related diseases, diabetes has been indicated to be closely associated with cellular aging and oxidative stress as well as inflammation [14–16]. Previous studies concerning the effect of Toc on diabetes have yielded conflicting results [17, 18]. Existing evidence comes from those with established diabetes or normal glucose tolerance (NGT), whereas no data were available in prediabetic individuals. Prediabetes is the earliest stage of diabetes (impaired glucose tolerance (IGT) and/or impaired fasting glucose (IFG)) which tends to progress to diabetes along with the loss of *β*-cell function, due in part to factors such as elevated glucose and lipid levels, inflammation, and oxidative stress [19]. Timely dietary intervention could reverse hyperglycemia or delay the onset of diabetes. Therefore, association between dietary intake of different Toc isoforms and markers of oxidative stress, inflammation, and cellular aging in prediabetic individuals may provide new insight into dietary intervention of prediabetes. However, it is still unknown how different glucose tolerance status affects the relationships between dietary intake of different Toc isoforms and cellular aging, oxidative stress, and inflammatory markers.

Accordingly, the authors investigated the cross-sectional associations of dietary exposure to different Toc isoforms with biomarkers of cellular aging, oxidative stress, and inflammation in a Chinese population with different glucose tolerance status.

## 2. Methods

### 2.1. Study Population

The study protocol has gained approval from the Ethics Committee of Peking Union Medical College Hospital. A total of 582 adult subjects aged between 18 and 81 residing in a Beijing suburb in China were recruited into the study from March 2014 to January 2015. All participants voluntarily provided written informed consents. The glucose tolerance status was classified according to the World Health Organization criteria [20]. The study participants were classified as NGT (*n* = 246), prediabetes (*n* = 174), and diabetes (*n* = 162). And the prediabetic individuals were further classified as IFG (*n* = 67), IGT (*n* = 58), and IFG+IGT (*n* = 49). Demographic and anthropometric data were collected by clinicians, including gender, age, weight, height, waist circumference (WC), hip circumference (HC), systolic blood pressure (SBP), and diastolic blood pressure (DBP).

### 2.2. Diet Assessment

As described in our previous publication [21], dietary data were collected with a 24-hour dietary recall, which were subsequently reviewed by a registered dietitian and analyzed using a nutrition calculation software developed by registered dietitians based on the Microsoft Office Access 2007 database. Intake estimates of key dietary components were calculated according to the China Food Composition (2004), including total Toc, *α*-Toc, *β*- and *γ*-Toc, and *δ*-Toc. Table S1 summarized the content of total Toc, *α*-Toc, *β*- and *γ*-Toc, and *δ*-Toc in different kinds of foods, which was provided as per 100 g of the edible portion (EP). Accordingly, the content of the different Toc isoforms per 1000 g food was calculated as follows: content per 100 g of EP × 10 × (EP/100).

### 2.3. Biochemical Analysis

The levels of fasting and 2-hour postload plasma glucose (FPG and PG120) during 75 g 2-hour oral glucose tolerance test were detected using glucose oxidase assay. The levels of fasting and 2-hour postload serum insulin (FINS and 2hINS) and C-peptide (FCP and 2hCP) were detected by chemiluminescent immunoassay. Glycosylated hemoglobin (HbA1c) was measured by high-performance liquid chromatography, and total cholesterol (TC), total triglyceride (TG), high-density lipoprotein cholesterol (HDL-C), low-density lipoprotein cholesterol (LDL-C), and uric acid (UA) were measured using an automatic analyzer.

### 2.4. LTL and mtDNAcn Assays

The details of LTL and mtDNAcn measurements have been elaborated in our previous publications [22, 23], both of which were determined by the real-time polymerase chain reaction method. LTL was calculated as the relative ratio of the telomere repeat copy number to the single copy number (T/S ratio) according to the monochrome multiplex quantitative polymerase chain reaction protocol. The relative mtDNAcn was adjusted by simultaneously measuring nuclear DNA.

### 2.5. Measurements of Biomarkers of Oxidative Stress and Inflammation

As previously described [22], levels of tumor necrosis factor (TNF-*α*), interleukine-6 (IL-6), superoxide dismutase (SOD) activity, 8-OHdG, and glutathione reductase (GR) were detected according to the manufacturer's instructions (Cloud-Clone Corp., Houston, USA).

### 2.6. Statistical Analysis

All the statistical analyses were conducted using SPSS 26.0 (IBM). Continuous variables were presented as mean ± standard deviation or median (interquartile range), and categorical variables were presented as percentages. Variables with nonnormal distribution were log transformed when necessary. Comparison of continuous variables among multiple groups were performed by one-way analysis of variance (ANOVA) or a nonparametric Mann-Whitney test with a post hoc Bonferroni test, where appropriate. Bivariate correlations were determined by Spearman's correlation analysis. Univariate and multivariate linear regression analyses were performed to quantify the relation among variables of interest. PROCESS macro Version 3.3 was applied to perform mediation model analysis among the variables statistically significant in univariate linear regression analysis. Mediation hypotheses were tested using a bias-corrected bootstrap method with 5000 samples to calculate 95% confidence intervals (95% CI). Statistical significance of mediating effect was set at zero not encompassed in the 95% CI. A 2-sided *p* value < 0.05 was considered statistically significant.

## 3. Results

### 3.1. Dietary Intake of Total Toc and Different Toc Isoforms Did Not Vary across Different Glucose Tolerance Status

**Figure 1:**
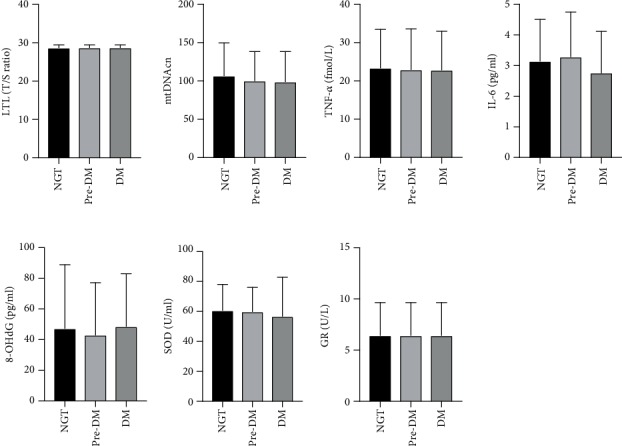
Comparison of cellular aging, oxidative stress, and inflammatory markers among different glucose tolerance status. The differences of cellular aging, oxidative stress, and inflammatory markers did not reach statistical significance (*p* > 0.05). Abbreviations: NGT—normal glucose tolerance; pre-DM—prediabetes; DM—diabetes mellitus; LTL—leukocyte telomere length; mtDNAcn—mitochondrial DNA copy number; TNF-*α*—tumor necrosis factor-*α*; IL-6—interleukine-6; SOD—superoxide dismutase; GR—glutathione reductase

**Figure 2:**
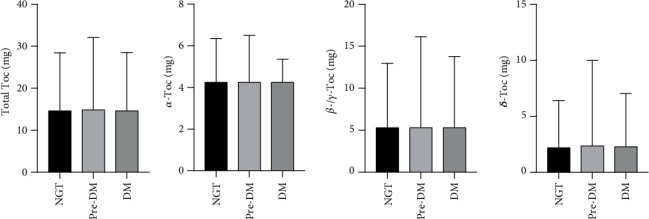
Comparison of dietary intake of total Toc and different Toc isoforms among different glucose tolerance status. The differences of dietary intake of total Toc and different Toc isoforms did not reach statistical significance (*p* > 0.05). Abbreviations: NGT—normal glucose tolerance; pre-DM—prediabetes; DM—diabetes mellitus; Toc—tocopherol(s)

As indicated in Table 1, compared with individuals with NGT, prediabetic and diabetic individuals had older age as well as higher body mass index (BMI), WC, HC, SBP, TC, TG, and LDL-C. Figures 1 and 2 presented the difference of cellular aging, oxidative stress, and inflammatory markers, as well as dietary intake of different Toc isoforms, respectively. It was shown that the differences among groups of cellular aging, oxidative stress, and inflammatory markers as well as dietary intake of different Toc isoforms did not reach statistical significance.

### 3.2. The Strongest Inverse Correlation of Dietary Non-*α*-Toc Isoform Intake with Level of TNF-*α* Was Observed in Prediabetic Individuals

To explore the association of dietary intake of different Toc isoforms with markers of cellular aging, oxidative stress, and inflammation in different glucose tolerance status, Spearman's correlation analysis was performed. As shown in Table 2, the non-*α*-Toc were inversely correlated with TNF-*α*. The strongest correlation was observed in prediabetic individuals (*r* = −0.33 for *β*-/*γ*-Toc, *r* = −0.37 for *δ*-Toc, *p* < 0.01), followed by individuals with NGT (*r* = −0.22 for *β*-/*γ*-Toc, *r* = −0.28 for *δ*-Toc, *p* < 0.01). In contrast, *α*-Toc was modestly positively correlated with TNF-*α* (*r* = −0.20, *p* < 0.01 in prediabetes, *r* = −0.19, *p* < 0.05 in NGT). However, in diabetic individuals, the authors failed to find a significant correlation of the dietary intake of different Toc isoforms with TNF-*α*. LTL was moderately, inversely correlated with *α*-Toc in prediabetic individuals (*r* = −0.30, *p* < 0.01), which was stronger than those in prediabetic and diabetic individuals (*r* = −0.19 and *r* = −0.18, respectively), whereas the positive correlations of LTL with non-*α*-Toc isoforms in different glucose tolerance status were modest (*r* = 0.11~0.20). Within the prediabetic individuals, the authors explored the associations between different Toc isoforms and cellular aging, oxidative stress, and inflammatory markers in individuals with IFG, IGT, and IFG+IGT, respectively (Table 3). The associations of different Toc isoforms with TNF-*α*, LTL, and mtDNAcn were the strongest in individuals with IFG. The dietary intake of non-*α*-Toc was strongly, negatively and *α*-Toc was moderately, positively correlated with the level of TNF-*α* in individuals with IFG (*r* = −0.55 for *δ*-Toc, *r* = −0.42 for *β*-/*γ*-Toc, and *r* = 0.35 for *α*-Toc, *p* < 0.01), while these correlations were significantly attenuated in individuals with IGT and IFG+IGT. The correlations of LTL/mtDNAcn with different Toc isoforms showed a similar variation trend across different glucose tolerance status (IFG > IGT ≈ IFG + IGT). The moderate-to-strong correlation between dietary intake of non-*α*-Toc and 8-OHdG was observed in individuals with IFG+IGT (*r* = −0.44 for *δ*-Toc, *r* = −0.38 for *β*-/*γ*-Toc, *p* < 0.01), which was remarkably weakened in individuals with isolated IFG or isolated IGT.

### 3.3. Dietary Intake of Non-*α*-Toc Was Independently Associated with mtDNAcn and Level of TNF-*α* in Prediabetic Individuals

As indicated in Table 4, univariate linear regression analysis revealed that dietary intake of non-*α*-Toc was significantly associated with LTL, mtDNAcn, and level of TNF-*α* in prediabetic individuals. After adjusting for covariates which possibly influenced the cellular aging, oxidative stress, and inflammatory markers including age, BMI, SBP, DBP, HbA1c, FPG, PG120, UA, TC, TG, HDL-C, and LDL-C in the multivariate linear regression model, non-*α*-Toc remained independently associated with mtDNAcn and level of TNF-*α* while the associations with LTL were substantially attenuated and statistically nonsignificant.

### 3.4. TNF-*α* Mediates the Protective Effect of Non-*α*-Toc Isoforms on Cellular Aging Markers in Prediabetic Individuals

**Figure 3:**
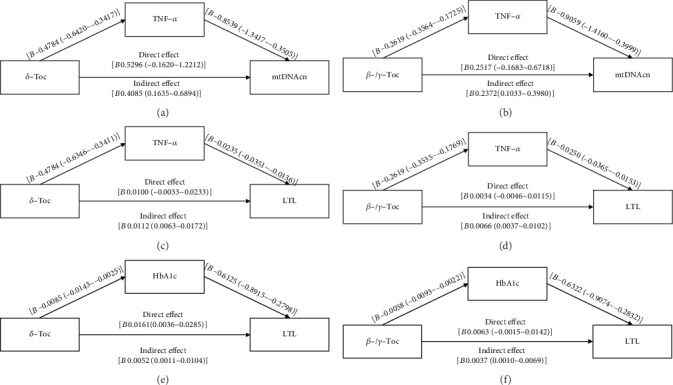
Mediation model of association among (a) *δ*-Toc, TNF-*α*, and mtDNAcn; (b) *β*-/*γ*-Toc, TNF-*α*, and mtDNAcn; (c) *δ*-Toc, TNF-*α*, and LTL; (d) *β*-/*γ*-Toc, TNF-*α*, and LTL; (e) *δ*-Toc, HbA1c, and LTL; and (f) *β*-/*γ*-Toc, HbA1c, and LTL in prediabetic individuals. Zero that is not included in the 95% CI represents statistical significance. Abbreviations: Toc—tocopherol(s); LTL—leukocyte telomere length; mtDNAcn—mitochondrial DNA copy number; TNF-*α*—tumor necrosis factor-*α*; HbA1c—glycosylated hemoglobin.

Considering that the negative association of non-*α*-Toc with cellular aging markers turned out to be statistically nonsignificant when adjusting for TNF-*α* as covariate (Table S2), and TNF-*α* has been reported to be involved in damage of TL [24] and mtDNAcn [13], the authors performed mediation model analysis to examine whether TNF-*α* mediated the protective effect of dietary intake of non-*α*-Toc on the cellular aging markers. As presented in Figures 3(a)–3(d), TNF-*α* mediated the protective effect of dietary intake of non-*α*-Toc on mtDNAcn (Figures 3(a) and 3(b)) and LTL (Figures 3(c) and 3(d)). And these mediation effects were uniquely observed in prediabetic individuals, while such mediation effects were statistically nonsignificant in individuals with NGT and established diabetes. Additionally, the negative association of non-*α*-Toc with cellular aging markers became statistically nonsignificant when adjusting for HbA1c as covariate (Table S2), and hyperglycemia reportedly accelerates telomere attrition [25]. The mediation model analysis showed that HbA1c also mediated the protective effect of the dietary intake of non-*α*-Toc on LTL (Figures 3(e) and 3(f)).

## 4. Discussion

To the authors' knowledge, this is the first study to explore the relationships of dietary intake of different Toc isoforms with cellular aging, oxidative stress, and inflammatory markers, and notably, the authors focused on the influence of different glucose tolerance status on these relationships in a population with consecutive glucose tolerance spectrum.

Interestingly, the authors observed that the inverse associations of dietary intake of non-*α*-Toc isoforms with the level of TNF-*α* varied substantially across different glucose tolerance status. The strongest association was observed in prediabetes, followed by NGT, while such association was abolished in established diabetes, which underlines the significance of timely dietary intervention for non-*α*-Toc isoform intake prior to the onset of established diabetes. The attenuation of the inverse association of non-*α*-Toc isoforms with TNF-*α* might be of critical importance in clinical practice. As a canonical proinflammatory cytokine, TNF-*α* plays an important role in the development of chronic inflammatory diseases. It has been demonstrated that persistent elevation of the level of TNF-*α* induces insulin resistance [26, 27] and pancreatic *β*-cell dysfunction [28] and thus exacerbates hyperglycemia, which could accelerate the onset of diabetes. Additionally, it was also reported to contribute to hyperglycemia-related disorders such as dyslipidemia [29], obesity, and cardiovascular diseases [30]. Therefore, our findings suggested that dietary intake of non-*α*-Toc might contribute to the delaying onset of diabetes and related metabolic disorders via suppression of the level of TNF-*α* in prediabetic individuals, which however, needs to be further validated by mechanistic studies. Emerging evidence suggested that non-*α*-Toc are superior to *α*-Toc in terms of antioxidant and anti-inflammatory properties [1–3]. It has been demonstrated in several studies that *δ*-Toc had the best anti-inflammatory effect, followed by *γ*-Toc and *α*-Toc [2]. In contrast, a few studies have yielded conflicting results that *δ*-Toc is responsible for a proinflammatory response promoted by reactive oxygen species and stress-activated Nrf2 and NF-*κ*B [31, 32]. This might be attributed to the different cell lines selected in different studies. Despite promising observations from in vitro and in vivo studies, human cohort and interventional studies regarding the effect of *α*-Toc or a mixture of *α*-Toc and *γ*-Toc in alleviating aging, oxidative stress, and inflammation yielded disappointing and equivocal outcomes [7, 33]. Human clinical investigations regarding the anti-inflammatory effect of *δ*-Toc are still lacking. However, in this study, the strongest association was found between *δ*-Toc and TNF-*α*. This finding suggests the important physiological and pharmacological discoveries of *δ*-Toc. More investigations regarding the biological function of *δ*-Toc should be encouraged.

Unexpectedly, within the prediabetic individuals, the inverse associations of non-*α*-Toc isoforms with the level of TNF-*α* also differed a lot (IFG > IGT ≈ IFG + IGT), suggesting that individuals with isolated IFG might benefit most from an appropriate increase of dietary non-*α*-Toc isoform intake in lowering the level of TNF-*α*. Current evidence on the prevention of diabetes relates to isolated IGT and IGT+IFG but not to isolated IFG [34]. Considering the contributions of persistent elevation of TNF-*α* to hyperglycemia [26–28], this preliminary finding might provide a novel dietary avenue for individuals with isolated IFG to alleviate hyperglycemia and delay the onset of diabetes, which might also be of immense clinical value. Nevertheless, large-scale prospective investigations are needed to further validate our cross-sectional findings, and experimental studies are necessary to elucidate the underlying mechanisms.

Additionally, the negative association of dietary intake of non-*α*-Toc with LTL and mtDNAcn in prediabetic individuals in linear regression analysis indicated that non-*α*-Toc might alleviate the shortening of LTL and the reduction of mtDNAcn, both of which have been previously demonstrated to be involved in the pathogenesis of diabetes [15, 16]. Currently, studies regarding the effect of Toc on LTL and mtDNAcn are still blank. Furthermore, in mediation model analysis, it was surprising to find that the mediation effect of TNF-*α* on non-*α*-Toc's protective effect on LTL and mtDNAcn was uniquely found in prediabetic individuals, suggesting that non-*α*-Toc might inhibit the cellular aging process via suppression of the level of TNF-*α* in prediabetic individuals. These findings might provide a novel insight into the possible regulation pathway by which non-*α*-Toc protect against cellular aging. Given the contributions of LTL and mtDNAcn to the pathogenesis of diabetes, these findings offer a potential avenue for prediabetic individuals to delay the onset of diabetes. However, further investigations are warranted.

In contrast to the well-established antioxidant effect of *α*-Toc in previous studies [3], the authors failed to find any protective effect of *α*-Toc on cellular aging, oxidative stress, and inflammatory markers. Unexpectedly, the authors found modest-to-moderate negative correlations of *α*-Toc with LTL and mtDNAcn, as well as modest positive correlations with TNF-*α*, which seemingly indicated procellular aging and proinflammatory effect of *α*-Toc. These results seem difficult to interpret, and thus further large-scale cohort studies are warranted to validate the current observations.

In the covariate correlation analysis, the authors failed to find any correlation between different Toc isoforms and oxidative stress markers including 8-OHdG, SOD, and GR in prediabetes. However, within individuals with combined IFG and IGT, a moderate-to-strong negative correlation was observed between non-*α*-Toc and level of 8-OHdG, a canonical product of DNA oxidation. Previous interventional studies were focused on *α*-Toc and have yielded conflicting results, with very limited investigations indicating a protective role of *α*-Toc in terms of alleviating DNA oxidative damage [7–9], while investigations regarding non-*α*-Toc were still lacking.

*α*-Toc is the major isoform in peanuts, almonds, and sunflower seeds, whereas *γ*-Toc is predominantly found in walnuts, pistachios, pecans, and sesame seeds. Despite the low content of *δ*-Toc, tomato seeds, rice germ, and soybean oil are rich sources of *δ*-Toc [1]. The present findings of the unique protective effect of non-*α*-Toc (especially for *δ*-Toc) on cellular aging and inflammation in prediabetic individuals suggest that those with prediabetes, especially for those with IFG, might benefit from an appropriate increase of dietary intake of non-*α*-Toc rather than *α*-Toc.

Several limitations need to be addressed. The limitations include (1) the lack of detection of the serum concentration of Toc which might make the results less convincing due to their highly different bioavailability; (2) the application of a 24-hour dietary recall which is relatively underrepresented for the daily dietary intakes of the participants; and (3) the lack of adjustment of potential dietary confounders such as fatty acids, phytosterols, or other lipid soluble dietary factors.

## 5. Conclusion

Collectively, the present results suggest that the dietary intake of non-*α*-Toc, especially for *δ*-Toc, might protect against cellular aging, which is mediated by TNF-*α* in prediabetic individuals. These observations highlight the possible benefit of dietary intake of non-*α*-Toc on alleviating inflammation and cellular aging. And the attenuation of the association between the dietary intake of non-*α*-Toc and TNF-*α* in established diabetes could suggest that timely intervention for dietary intake of non-*α*-Toc in the prediabetes stage might be critical for the prevention or delay of diabetes onset. However, further mechanistic studies are warranted to validate these observations.

## Figures and Tables

**Table 1:** Difference of clinical characteristics of individuals with different glucose tolerance status.

	NGT (*n* = 246)	Pre-DM (*n* = 174)	DM (*n* = 162)	*p* value
Gender (M/F)	168/78	106/68	103/59	0.277
Age (years)	49.41 ± 11.64	56.41 ± 10.68^a^	56.85 ± 10.08^a^	<0.001
BMI (kg/m^2^)	25.23 ± 3.58	26.63 ± 3.62^a^	26.40 ± 3.81^a^	<0.001
WC (cm)	85.19 ± 9.53	88.77 ± 9.00^a^	88.50 ± 9.77^a^	<0.001
HC (cm)	90.58 ± 9.62	94.42 ± 8.99^a^	93.80 ± 12.44^a^	<0.001
SBP (mmHg)	124.55 ± 17.07	128.75 ± 15.89^a^	132.19 ± 19.08^a^	<0.001
DBP (mmHg)	75.96 ± 9.41	75.97 ± 10.21	76.81 ± 10.48	0.654
HbA1c (%)	5.41 ± 0.53	5.71 ± 0.38^a^	7.25 ± 1.57^a,b^	<0.001
FPG (mmol/L)	5.53 (5.25, 5.81)	6.22 (5.87, 6.50)^a^	8.41 (7.26, 10.17)^a,b^	<0.001
2hPG (mmol/L)	6.11 (5.17, 7.15)	8.04 (6.78, 9.30)^a^	15.43 (11.71, 19.42)^a,b^	<0.001
FINS (*μ*IU/mL)	8.93 (6.62, 12.23)	9.77 (7.24, 13.84)	11.38 (7.38, 17.55)^a^	<0.001
2hINS (*μ*IU/mL)	34.70 (19.36, 52.84)	54.47 (36.07, 79.47)^a^	46.92 (25.94, 86.65)^a^	<0.001
FCP (ng/mL)	1.20 (0.94, 1.55)	1.38 (1.15, 1.91)^a^	1.44 (0.96, 2.02)^a^	<0.001
2hCP (ng/mL)	4.62 (3.33, 5.89)	6.29 (4.66, 8.18)	5.19 (3.25, 7.40)^a,b^	<0.001
UA (*μ*mol/L)	280.99 ± 76.93	308.66 ± 86.51^a^	289.18 ± 75.87	0.002
TC (mmol/L)	5.30 ± 0.95	5.61 ± 1.05^a^	5.46 ± 1.16	0.013
TG (mmol/L)	1.22 (0.82, 1.61)	1.53 (1.08, 2.28)^a^	1.72 (1.17, 2.37)^a^	<0.001
HDL-C (mmol/L)	1.32 ± 0.27	1.28 ± 0.26	1.24 ± 0.27^a^	0.023
LDL-C (mmol/L)	2.69 ± 0.71	2.96 ± 0.69^a^	2.87 ± 0.73^a^	<0.001

^a^ *p* < 0.05 compared with NGT. ^b^*p* < 0.05 compared with pre-DM. Abbreviations: NGT—normal glucose tolerance; pre-D—prediabetes; DM—diabetes mellitus; BMI—body mass index; WC—waist circumference; HC—hip circumference; SBP—systolic blood pressure; DBP—diastolic blood pressure; HbA1c—glycosylated hemoglobin; FPG—fasting plasma glucose; 2hPG—2 h postload plasma glucose; FINS—fasting insulin; 2hINS—2 h postload insulin; FCP—fasting C-peptide; 2hCP—2 h postload C-peptide; UA—uric acid; TC—total cholesterol; TG—total triglyceride; HDL-C—high-density lipoprotein cholesterol; LDL-C—low-density lipoprotein cholesterol.

**Table 2:** Spearman's correlations of different Toc isoforms with markers of cellular aging, oxidative stress, and inflammation in various glucose tolerance status.

	Total Toc	*α*-Toc	*β*-/*γ*-Toc	*δ*-Toc
NGT (*n* = 246)				
LTL	0.15^∗^	-0.19^∗∗^	0.16^∗^	0.18^∗∗^
mtDNAcn	0.11	-0.12	0.13^∗^	0.13^∗^
TNF-*α*	-0.21^∗∗^	0.20^∗∗^	-0.22^∗∗^	-0.28^∗∗^
IL-6	-0.03	0.02	-0.04	-0.03
8-OHdG	0.04	-0.04	0.04	0.06
SOD	-0.10	0.24^∗∗^	-0.13^∗^	-0.23^∗∗^
GR	0.00	0.03	0.03	0.01
Pre-DM (*n* = 174)				
LTL	0.11	-0.30^∗∗^	0.14	0.16^∗^
mtDNAcn	0.04	-0.19^∗^	0.06	0.08
TNF-*α*	-0.32^∗∗^	0.19^∗^	-0.33^∗∗^	-0.37^∗∗^
IL-6	0.02	0.15^∗^	-0.01	-0.05
8-OHdG	-0.11	0.07	-0.12	-0.10
SOD	-0.13	0.11	-0.14	-0.17^∗^
GR	0.06	0.08	0.05	0.03
DM (*n* = 162)				
LTL	0.13	-0.18^∗^	0.14	0.20^∗^
mtDNAcn	-0.05	-0.02	-0.05	-0.05
TNF-*α*	-0.08	0.10	-0.12	-0.11
IL-6	0.09	0.10	0.07	0.09
8-OHdG	-0.11	-0.10	-0.10	-0.05
SOD	-0.05	0.15	-0.07	-0.06
GR	0.03	-0.03	0.02	0.04

^∗^ *p* < 0.05 and ^∗∗^*p* < 0.01. Abbreviations: NGT—normal glucose tolerance; pre-DM—prediabetes; DM—diabetes mellitus; Toc—tocopherol(s); LTL—leukocyte telomere length; mtDNAcn—mitochondrial DNA copy number; TNF-*α*—tumor necrosis factor-*α*; IL-6—interleukine-6; SOD—superoxide dismutase; GR—glutathione reductase.

**Table 3:** Spearman correlations of different Toc isoforms with markers of cellular aging, oxidative stress, and inflammation in different groups of prediabetic individuals.

	Total Toc	*α*-Toc	*β*-/*γ*-Toc	*δ*-Toc
IFG (*n* = 67)				
LTL	0.23	-0.39^∗∗^	0.26^∗^	0.27^∗^
mtDNAcn	0.18	-0.37^∗∗^	0.20	0.39^∗∗^
TNF-*α*	-0.39^∗∗^	0.35^∗∗^	-0.42^∗∗^	-0.55^∗∗^
IL-6	-0.02	0.17	-0.10	-0.11
8-OHdG	0.12	0.04	0.14	0.13
SOD	-0.05	-0.04	-0.04	-0.10
GR	0.02	-0.02	0.04	0.01
IGT (*n* = 58)				
LTL	0.05	-0.18	0.06	0.09
mtDNAcn	0.10	-0.12	0.12	0.02
TNF-*α*	-0.26^∗^	-0.06	-0.27^∗^	-0.21
IL-6	0.05	0.07	-0.01	-0.00
8-OHdG	-0.20	0.09	-0.24	-0.08
SOD	-0.12	0.12	-0.09	-0.11
GR	0.11	0.07	0.06	0.03
IFG+IGT (*n* = 49)				
LTL	0.00	-0.35^∗^	-0.00	0.07
mtDNAcn	-0.22	-0.10	-0.20	-0.27
TNF-*α*	-0.25	0.24	-0.25	-0.23
IL-6	0.04	0.20	0.07	-0.01
8-OHdG	-0.40^∗∗^	0.12	-0.38^∗∗^	-0.44^∗∗^
SOD	-0.28	0.21	-0.31^∗^	-0.25
GR	0.02	0.21	0.03	0.08

^∗^ *p* < 0.05 and ^∗∗^*p* < 0.01. Abbreviations: Toc—tocopherol(s); IFG—impaired fasting glucose; IGT—impaired glucose tolerance; LTL—leukocyte telomere length; mtDNAcn—mitochondrial DNA copy number; TNF-*α*—tumor necrosis factor-*α*; IL-6—interleukine-6; SOD—superoxide dismutase; GR—glutathione reductase.

**Table 4:** Univariate and multivariate linear regression analyses of different Toc isoforms with cellular aging, oxidative stress, and inflammatory markers in prediabetic individuals.

	LTL		mtDNAcn		TNF-*α*		Log2 (IL-6)		Log2 (8-OHdG)		SOD	
	*β*	*p*	*β*	*p*	*β*	*p*	*β*	*p*	*β*	*p*	*β*	*p*
Univariate linear regression model												
Total Toc	0.001	0.511	0.131	0.220	-0.110	<0.001	-0.001	0.700	-0.005	0.155	-0.012	0.789
*α*-Toc	-0.0216	0.086	-0.787	0.215	0.301	0.089	0.018	0.269	0.019	0.330	0.303	0.261
*β*-/*γ*-Toc	0.010	0.015	0.489	0.018	-0.262	<0.001	-0.003	0.596	-0.009	0.142	-0.050	0.573
*δ*-Toc	0.021	0.001	0.938	0.005	-0.478	<0.001	-0.009	0.286	-0.014	0.166	-0.113	0.432
Multivariate linear regression model												
Total Toc	<0.001	0.978	0.144	0.186	-0.111	<0.001	-0.002	0.378	-0.005	0.178	-0.007	0.861
*α*-Toc	-0.016	0.188	-0.868	0.187	0.291	0.109	0.019	0.225	0.018	0.382	-0.551	0.044
*β*-/*γ*-Toc	0.005	0.222	0.559	0.010	-0.254	<0.001	-0.004	0.427	-0.009	0.174	-0.024	0.779
*δ*-Toc	0.011	0.063	1.051	0.003	-0.458	<0.001	-0.009	0.295	-0.016	0.142	-0.065	0.634

Multivariate linear regression model adjusted for age, BMI, SBP, DBP, HbA1c, FPG, PG120, UA, TC, TG, HDL-C, and LDL-C as covariates. Abbreviations: Toc—tocopherol(s); LTL—leukocyte telomere length; mtDNAcn—mitochondrial DNA copy number; TNF-*α*—tumor necrosis factor-*α*; IL-6—interleukine-6; SOD—superoxide dismutase; GR—glutathione reductase.

## Data Availability

The SPSS Statistics data used to support the findings of this study are available from the corresponding author upon request.

## Abbreviations

Toc: tocopherol(s)
8-OHdG: 8-hydroxy-2deoxyguanosine
LTL: leukocyte telomere length
mtDNAcn: mitochondrial DNA copy number
NGT: normal glucose tolerance
IGT: impaired glucose tolerance
IFG: impaired fasting glucose
WC: waist circumference
HC: hip circumference
SBP: systolic blood pressure
DBP: diastolic blood pressure
EP: edible portion
FPG: fasting plasma glucose
2hPG: 2 h postload plasma glucose
FINS: fasting insulin
2hINS: 2 h postload insulin
FCP: fasting C-peptide
2hCP: 2 h postload C-peptide
HbA1c: glycosylated hemoglobin A1c
TC: total cholesterol
TG: total triglyceride
HDL-C: high-density lipoprotein cholesterol
LDL-C: low-density lipoprotein cholesterol
UA: uric acid
TNF-*α*: tumor necrosis factor-*α*
IL-6: interleukine-6
SOD: superoxide dismutase
GR: glutathione reductase
95% CI: 95%confidence intervals
BMI: body mass index
DM: diabetes mellitus.

## Supplementary Materials

Supplementary 1 Table S1: summary of content of total tocopherols and different tocopherol isoforms in different kinds of foods. The data represented the content per 100 g of the edible portion.

Supplementary 2 Table S2: multivariate linear regression model analysis of non-*α*-Toc as an independent variable.
