# Supplementary material for: Potential Protective Effect of Dietary Intake of Non-α-Tocopherols on Cellular Aging Markers Mediated by Tumor Necrosis Factor-α in Prediabetes: A Cross-Sectional Study of Chinese Adults
Source: Oxid Med Cell Longev. 2020 May 15;2020:7396801. doi: 10.1155/2020/7396801 (PMC7245674; doi:10.1155/2020/7396801)
Supplement: Supplementary 1 — Table S1: summary of content of total tocopherols and different tocopherol isoforms in different kinds of foods. The data represented the content per 100 g of the edible portion. [file 7396801.f1.docx]

Table S1. Multivariate linear regression model analysis of non-α-Toc as an independent variable.

|  | β coefficient | Standard error | *P* value |
| --- | --- | --- | --- |
| **Dependent variable: LTL** | | | |
| TNF-α | -0.023 | 0.005 | 0.000 |
| δ-Toc | 0.010 | 0.007 | 0.138 |
| **Dependent variable: LTL** | | | |
| TNF-α | -0.025 | 0.005 | 0.000 |
| β-/γ-Toc | 0.003 | 0.004 | 0.401 |
| **Dependent variable: mtDNAcn** | | | |
| TNF-α | -0.854 | 0.282 | 0.003 |
| δ-Toc | 0.530 | 0.350 | 0.132 |
| **Dependent variable: mtDNAcn** | | | |
| TNF-α | -0.906 | 0.278 | 0.001 |
| β-/γ-Toc | 0.252 | 0.213 | 0.238 |
| **Dependent variable: LTL** | | | |
| HbA1c | -0.612 | 0.141 | 0.000 |
| δ-Toc | 0.016 | 0.006 | 0.012 |
| **Dependent variable: LTL** | | | |
| HbA1c | -0.632 | 0.143 | 0.000 |
| β-/γ-Toc | 0.006 | 0.004 | 0.110 |

Abbreviations: LTL, leukocyte telomere length; TNF-α, tumor necrosis factor-α; Toc, tocopherol(s); mtDNAcn, mitochondrial DNA copy number; HbA1c, glycosylated hemoglobin A1c
